# Relationship of Salivary Alpha Amylase and Cortisol to Social Anxiety in Healthy Children Undergoing Laboratory Pain Tasks

**DOI:** 10.4172/jcalb.1000129

**Published:** 2014-04-23

**Authors:** Laura A Payne, Leah C Hibel, Douglas A Granger, Jennie C I Tsao, Lonnie K Zeltzer

**Affiliations:** 1 David Geffen School of Medicine at UCLA, Los Angeles, CA, USA; 2 University of California, Davis, CA 95616, USA; 3 Arizona State University, 11 North Central Avenue, Phoenix, AZ 85004, USA

**Keywords:** Alpha amylase, Cortisol, Social anxiety, Anxiety, stress, Children, Youth, Pain

## Abstract

**Objective:**

Salivary alpha amylase (sAA) has been shown to be a sensitive and reliable marker of the autonomic nervous system (ANS) response to stress. A link between sAA, cortisol, and social/evaluative stress has been established in youth, but little is known about these relationships in response to other stressors in children, and how social anxiety might moderate these relationships. The current study explored the associations among sAA and salivary cortisol responses to laboratory pain tasks and self-reported social anxiety symptoms in a sample of healthy children.

**Method:**

Two hundred thirty-one children (114 girls; 49.4%) with a mean age 12.68 years (SD=3.0; range 7–18) participated in the study. Participants completed self-report questionnaires prior to undergoing a series of laboratory pain tasks involving cold, pressure, and heat pain. Saliva samples were collected upon arrival to the laboratory (pre-task), following the completion of the pain tasks (post-task1), and 20 minutes after the completion of the pain tasks (post-task2).

**Results:**

Demographic factors (age, sex, pubertal stage) did not predict either sAA or cortisol levels. However, children reporting higher levels of social anxiety demonstrated significantly higher sAA but not cortisol levels across three salivary collection times, compared to children reporting lower levels of social anxiety. Further, it does not appear that reduced state levels of anxiety before or during the tasks buffer this relationship.

**Conclusion:**

These data highlight the possibility of identifying biomarkers of stress that are consistent across time and developmental stage. sAA appears to be a marker of stress response in children with self-reported social anxiety. There may also be a potentially unique relationship of sAA to stress in this population. In addition, sAA may reflect stable individual differences in levels of ANS arousal and may be a useful biomarker for identifying children at risk for stress.

## Introduction

National estimates reveal that approximately 5.5% of children and adolescents suffer various degrees of social anxiety [1]. Fear and avoidance of social situations in childhood is associated with negative developmental outcomes such as depression, anxiety and school refusal. Studies with adults suggest that individual differences in social anxiety may be accompanied by heightened physiological responses to social stress, threat, and challenge [2]. Theorists speculate that individual differences in physiological reactivity and regulation in these circumstances provide clues as to how everyday concerns and worries develop over time into patterns of symptoms with the potential to impact psychosocial adjustment. Surprisingly, however, the depth of our knowledge about the association between social anxiety and physiological reactivity in youth is very shallow [3]. In this study we report a pattern of findings that begins to address this information gap.

Developmental science has championed salivary cortisol to operationalize individual differences in the activity of the hypothalamic-pituitary-adrenal (HPA) axis. Recently, studies have shown salivary alpha amylase (sAA), a surrogate marker of autonomic nervous system (ANS) activity, to have similar utility in studies of biosocial processes, particularly those related to stress and anxiety [4]. Specifically, increased cortisol levels in response to social stress and fear has been well-documented [5], and heightened sAA has been related to both generalized social anxiety disorder [2] and fear [6].

While few studies have examined the relationship between social anxiety and stress physiology in childhood and adolescence, studies exploring HPA and ANS responses to social/evaluative stressors in children and adolescents suggest a relationship may exist. Regarding cortisol responses, one study demonstrated elevated levels of cortisol to both a social/evaluative stress test (the Trier Social StressTest) and attending school in adolescent girls both with and without social anxiety [7]. While no difference between girls with and without social anxiety was found, it could be that cortisol alone is not a sufficient marker of individual differences in social anxiety. Recent research has therefore turned to sAA as a potential marker for social stress reactivity in younger populations. For example, Stroud et al. [8], demonstrated developmental differences in sAA reactivity to social stress, with elevated sAA responses in adolescents experiencing peer rejection as compared to children. In addition, sAA levels in responses to a social stressor (either performance or peer rejection) have also been shown to predict general anxiety in youth [9].

Further, little is known about sAA and cortisol reactivity to nonsocial/evaluative stressors in youth. Yet, emerging evidence now supports the concept of sAA as a marker of stable, individual differences in response to stressors across a variety of contexts [10], with some evidence pointing to sAA increases in response to both high arousal negative emotions (e.g., anger, stress, worry) and high arousal positive emotions (e.g., feeling strong, active, excited) [11]. Together these results suggest that sAA may share a unique relationship with emotional states that may be pronounced under general stress that do not include a social/evaluative component. Determining the relationship of sAA responses to other types of stressors is clearly the next step to characterizing the impact of stress on physiological responses.

The current study explored the relationship between social anxiety and stress physiology (i.e., sAA and cortisol) in a sample of healthy children and adolescents undergoing a series of non-social/evaluative stressors (laboratory pain tasks). No existing work has yet explored sAA and cortisol responses to a laboratory pain stressor; although based on prior research, we hypothesized that (1) both sAA and cortisol levels would be elevated in response to the laboratory tasks [12]; however, children with self-reported higher levels of social anxiety will show an elevated sAA response as compared to those with self-reported low social anxiety, reflecting the possibility that sAA will capture individual differences in the stress response. In addition, we will examine a series of interactions including pubertal stage, sex, collection time of day, and anticipatory and task anxiety to help disentangle the relationship between social anxiety and stress physiology. Based on research suggesting that the sAA response is sensitive to puberty [8,13], we hypothesize that pubertal stage will moderate the sAA/cortisol relationship in this sample of healthy youth. Further, we will explore the potential for reduced state anxiety before and during the task to buffer the relationship between stress physiology and social anxiety.

## Methods

### Participants

The University of California, Los Angeles Institutional Review Board (IRB) and the IRB recruitment sites approved all recruitment and study procedures. All procedures were carried out with adequate understanding, written consent of parents, and written assent of children. Participants were recruited through mass mailings, posted advertisements, and classroom presentations. Participants were part of a larger study exploring laboratory pain responses in healthy children [14–16].

Inclusion criteria were as follows: self-reported healthy children and adolescents, aged 8 to 18 years. Exclusion criteria were: (1) current acute or chronic illness; (2) developmental delay or impairment that would preclude understanding of study procedures or participation in pain induction procedures; or (3) daily use of opioid medication. The use of analgesic medication on the day of study participation was prohibited. No other exclusion criteria were required of participants.

The final sample consisted of 231 children (114 girls; 49.4%), mean age 12.68 years (SD=3.0; range 7–18). Approximately half the sample (46.3%; n=107) self-identified as being in early puberty, half of which were female (54 girls, 49.5%). The racial/ethnic composition of the sample was 37.7% Caucasian (n=87), 21.6% Hispanic (n=50), 12.1% African American (n=28), 5.2% Asian/Pacific Islander (n=12), and 16% other (n=37). Racial/ethnic data were unavailable for 17 participants.

### Measures

#### Multidimensional anxiety scale for children [MASC, 17]

The MASC is a 39-item measure of anxiety symptoms consisting of four 4-point subscales: social anxiety, harm avoidance, separation anxiety, and anxiety over physical symptoms. For the current study, only the social anxiety subscale was used, as this measure has shown good convergent validity with other measures of social anxiety and is a useful initial assessment tool in samples of non-referred youth [18]. The t-score cut off of 65 (indicating possible clinically significant anxiety) for the social anxiety subscale translates to a raw score of 19 for females and 18 for males [17].

#### Pubertal stage

Schematic drawings, including appropriate written descriptions of 5 stages of secondary sexual characteristics on 2 separate dimensions (female breasts and pubic hair, male genitalia and pubic hair) based on Tanner’s Sexual Maturity Scale, were administered for self-report of pubertal status [19,20]. Early puberty was operationalized as Tanner Stages I–III, while later puberty was defined as Tanner Stages IV–V.

#### Anxiety

Anticipatory anxiety and anxiety during the task were assessed through a vertical sliding Visual Analog Scale (VAS) immediately after each trial. The VAS is brief, easily understood, and sensitive to changes in pain. With excellent psychometric properties [21], it can be used by children included in the current study (age range 8–18 years) [22]. Previous research used the VAS to rate pain in children in laboratory pain tasks [23].

The VAS was anchored with 0 at the bottom and 10 at the top. The scale also had color cues, graded from white at the bottom to dark red at the top, as well as a neutral face at the bottom and a negative facial expression at the top. Participants were given these instructions regarding the use of VAS: “This scale is like a thermometer, only rather than measure your temperature; we will use it to measure your feeling or mood. The white color on the bottom represents the lowest values and the dark red at the very top represents the highest values for a particular feeling. By using this thermometer, you’ll let me know how much pain or discomfort you feel. You will do this by sliding the bar up and down on the colors until you get to the shade that equals how you feel.”

Prior to each trial of each task, participants were asked to use the VAS to answer the question “how nervous, afraid, or worried are you about the upcoming task?” (anticipatory anxiety). Following each trial of each task, participants used the VAS to answer “how nervous, afraid, or worried were you during the task?” (task anxiety). Within each task, trial scores were averaged to produce a single score for each anticipatory anxiety and task anxiety for each task.

### Procedures

Laboratory sessions were primarily conducted in the afternoon; however, there was some variation in the time of day that participants participated in the study, so the time of day was explored as a potential covariate in the analyses to control for these variations. In brief [14–16], after completing questionnaires, children participated in a series of tasks that involved self-report and behavioral responses to cold, pressure, and heat tasks, with the order of tasks counterbalanced. The three tasks involved immersing the hand in cold water, applying pressure using a weighted lucite point to the finger, and holding the forearm over a radiant heat diode. Each task had a uniformed ceiling. Before the start of each trial, participants were informed that they would experience moderate sensation that might eventually be perceived as pain. They were instructed to continue with each task for as long as they could, and to remove their finger or arm from the apparatus at any time during the procedures if it became too uncomfortable or painful. All tasks were extensively pilot-tested with volunteers in the targeted age range.

#### Determination of salivary analytes

Saliva samples were obtained from participants after they entered the laboratory (pre-task), after the completion of all pain tasks (post-task1), and at the end of the session 20 minutes after the previous assessment (post-task2). The timing between pre-task and post-task1 samples varied to some degree across participants (depending on how long each required to complete the questionnaires and pain tasks; M=1 hr 13 mins, SD=14 mins, range: 17 mins – 2 hrs 35 mins), although the timing between post-task1 and post-task2 samples was standardized (20 minutes). Unstimulated saliva samples were collected by instructing the participants to gently mouth cotton rolls for 3 minutes. Following collection, samples were frozen at −80°Celsius. Cortisol was analyzed in duplicate using a commercially available enzyme immunoassay without modification to the manufacturer’s protocol (Salimetrics, State College, PA), range of sensitivity from .007–3.0 μg/dL, and intra- and inter-assay coefficients of variation less than 5 and 10% respectively. Salivary cortisol data are expressed in μg/dL.

sAA was measured using a kinetic reaction assay that employs a chromagenic substrate, 2-chloro-p-nitrophenol, linked to maltotriose [Salimetrics, state College, PA; see 4 for a detailed explanation]. Intraassay variation (CV) computed for the mean of 30 replicate tests was less than 7.5%. Interassay variation computed for the mean of average duplicates for 16 separate runs was less than 6%. sAA data are expressed in U/mL.

## Analytical Strategy

First, descriptive statistics exploring the demographic characteristics associated with social anxiety are presented. Second, descriptives overviewing the changes in cortisol and sAA levels across the Sampling Time period (pre-task, post-task1, and post-task2) are provided. Then, mixed model ANCOVAs were computed to identify potential demographic (age, pubertal stage, sex) factors associated with cortisol and sAA levels, and change across the pain tasks. The omnibus ANOVA design included Sampling Time as a repeated measure and Social Anxiety as the independent variable, and cortisol, and sAA and as the dependent variables. Analyses were conducted on continuous variables; high and low social anxiety groups were determined based on median splits and are for comparison only. Potential moderators (pubertal stage, sex, collection time of day, anticipatory and task anxiety) were then explored. To correct for the skew present in the cortisol (range: 4.41 to 1.78) and sAA (range: 1.63 to 1.93) values, a log transformation was applied to the cortisol measurements and a square root transformation was applied to the sAA. Both transformations fixed the skew resulting in skew statistics within normal range (cortisol range: −.19 to .24; sAA range .608 to .728). Outliers three standard deviations beyond the mean were trimmed. For ease of interpretation raw scores are reported.

## Results

### Social anxiety

The majority of the sample (84.1%) was below the t-score cut-off for social anxiety subscale of the MASC (M=9.4, SD=5.7, range=0–26). Norms for the social anxiety subscale by age and sex are reported in Table 1. Females were not more likely to have social anxiety scores above the cut-off Χ(1)=.10, p = ns. Similarly, older Χ(2)=2.10, p = ns or more developed Χ(1)=1.10, p=ns children were not more likely to have clinical levels of social anxiety. Further, neither age r(242)=.01, p=ns, nor pubertal status r(242)=.08, p=ns were related to scores on the social anxiety subscale. Finally, scores on the nine items making up the social anxiety scale did not differ based on age (p’s from .11 to .99), however, girls were more likely to report being afraid of being thought of as stupid t(241)=1.97, p=.05 and worrying people would laugh at them t(241)=2.93, p<.01.

### Predictors of sAA/cortisol

Overall, there was no significant change in sAA from pre- to posttask2, F (2, 232) = 0.96, p=ns. Cortisol, on the other hand, changed across the session exhibiting a decrease from the pre task collection to the post-task1 collection, but remained stable from the post-task1 to the post-task2 collection, F (1, 232)=142.66, p<.0001 (Table 2).

**Figure 1:**
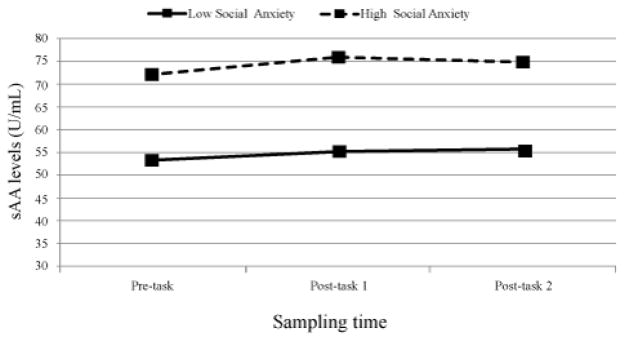
Children reporting higher levels of social anxiety exhibited higher levels of sAA at all three collection points (p<.01). Lines represent children below (Low: N=123) and above (High: N=108) the median level of social anxiety.

Collection time of day was negatively related, F (1,233)=73.52, p<. 0001, while pubertal stage was positively related F (1,228)=19.55, p<. 0001 (Early puberty: M=0.10 ug/mL; SD=0.06; Late puberty: M=0.14 ug/mL, SD=0.10) to cortisol levels. Sex (Male/Female) was not related to cortisol levels, and neither collection time of day, pubertal stage, nor sex were related to cortisol change across the tasks (ps ranging from 0.12 to 0.56). Neither collection time of day, sex, nor pubertal stage were related to sAA levels (ps ranging from 0.16 to .59) or sAA change across the pain tasks (ps ranging from 0.43 to 0.97). The levels F (1, 233)=1.60, p=ns and trajectories of sAA and cortisol were not related F (2, 233)=0.04, p=ns. Social anxiety was found to be a significant predictor of sAA levels across the tasks F (1, 231)=6.65, p=.01, revealing children who rated themselves as having higher levels of social anxiety exhibited significantly higher sAA levels at each of the three salivary collection points (low social anxiety: 53.40, 55.27, 55.41; high anxiety: 72.02, 76.05, 74.85 U/mL). See Figure 1 for a graph of these values.

### Potential moderators

Demographic factors were then examined as possible moderators of the relationship between social anxiety, and sAA and cortisol. Neither collection time of day, sex, nor pubertal stage interacted with social anxiety to predict sAA levels (ps ranging from 0.15 to .65) or sAA change across the pain tasks (ps ranging from 0.25 to 0.31). Similarly, these variables did not interact with social anxiety to predict cortisol levels (ps ranging from 0.22 to .75) or cortisol change (ps ranging from 0.45 to .67). Lastly, we examined if reports of anticipatory anxiety or task anxiety buffered the relationship between social anxiety and stress physiology. Interactions of social anxiety and anticipatory anxiety to the task did not predict cortisol F (1, 230)=2.56, ns or sAA levels F (1, 229)=0.85, ns or cortisol F (1, 230)=0.86, ns or sAA change across the tasks F (1, 229)=0.80, ns. Similarly, interactions social anxiety and task anxiety did not predict cortisol F (1, 227)=0.05, ns, or sAA levels F (1, 226)=0.40, ns, or cortisol F (1, 227)=0.33, ns or sAA change across the task F (1, 226)=0.62, ns.

## Discussion

Children reporting higher levels of social anxiety demonstrated significantly higher levels of sAA across the laboratory session compared with children reporting lower levels of social anxiety. These data mirror findings in adults with generalized social phobia [2] and suggest that sAA may be an important marker of stress in individuals sensitive to social evaluation. Interestingly, social anxiety was not related to cortisol levels or cortisol change across the tasks. Our data suggest a potentially unique contribution of sAA (and the ANS) to understanding the stress response in children with social anxiety. In addition, our results demonstrated that social anxiety was related to sAA at all three collection timepoints, suggesting that sAA is a potential marker of stable, trait-based differences [24].

Furthermore, our data highlight the possibility of identifying biomarkers of stress that are consistent across time and developmental stage, given our results showing the lack of association with sampling time, age, sex, or pubertal stage. Unlike cortisol which captures acute stress responses, sAA may reflect stable individual differences in levels of ANS arousal and thus general stress reactivity. Our data support findings from Allwood et al. [9], suggesting that sAA levels in response to a stressor are associated with trait-based levels of anxiety, given reports of anxiety did not moderate the social anxiety-sAA relationship. This idea is consistent with other psychophysiological measures of the ANS, and emerging evidence that a significant amount of the variance in sAA is accounted for by individual differences in ANS set-points [8]. In support of this notion, there was no interaction of social anxiety and anticipatory anxiety or task anxiety to sAA or cortisol levels, or sAA or cortisol change. This also suggests that taskspecific anxiety may not be related to increased cortisol or sAA responses, and instead the elevated sAA responses only evident in participants with high social anxiety may reflect a trait-based difference.

Clinically, our data suggest the importance of both assessing and treating social anxiety in children and adolescents, as they may be at risk for chronically elevated ANS arousal. When children with high social anxiety are exposed to stressors, it is important that an intervention target the social anxiety, which may allow their ANS to recover. Interventions such as cognitive-behavioral therapy (CBT) may be a useful tool for clinicians to help children and adolescents reduce their overall levels of stress.

However, a number of important caveats to the study should be noted. Results may be different in children diagnosed with social anxiety disorder. As previously stated, the current study data were drawn from a larger study exploring children’s responses to laboratory pain, so different tasks that reflect other types of stressors may produce a different pattern of results. It is also possible that the three time points at which saliva samples were collected were not sufficient to capture additional variations in sAA levels. We also did not gather data on the use of contraceptives or other hormonal medications, which may have skewed results. Recent criticisms have highlighted methodological issues (i.e., use of absorbent cotton roll for collecting saliva) in sAA research and raised concerns about the validity and reliability of sAA as a biomarker [25]. However, our use of standardized collection procedures to reduce variability in flow rate at least partially addressed this issue. Future research should continue to explore sAA levels in response to stressors in a variety of child and adolescent populations and across different contexts.

Although it is not clear whether this pattern would remain robust across settings, it nonetheless raises important questions about how we understand and measure state- and trait-based biomarkers of stress. Biomarkers of early stress vulnerability might inform how childhood stress is related to adult diseases and conditions, under the theory that the body’s cumulative efforts to adapt to acute stress could eventually affect various body systems. Characterization of the stress response in children could identify individuals at risk for ongoing stress and future psychopathology, which suggests the potential for early intervention [26].

## Figures and Tables

**Table 1:** Non-clinical normative means for social anxiety subscale of the MASC by age group and sex.

Age group	Female M(SD)	Male M(SD)
8–11 years	10.28(5.73)	9.02(5.85)
12–15 years	10.03(5.59)	8.32(5.38)
16–19 years	9.79(5.54)	8.07(5.67)

**Table 2:** Means and standard deviations for salivary alpha amylase (sAA) and cortisol levels across the laboratory session for the entire sample

	Pre-task M (SD)	Post-task 1M (SD)	Post-task2 M (SD)
**sAA (U/mL)**	62.14 (48.51)		64.50 (50.91)
**Cortisol (ug/mL)**	0.15 (0.14)	0.09 (0.06)*	0.11 (0.13)*

Note: sAA=salivary alpha amylase.

* denotes significant change (p<.01) from Pre-task levels.

## References

[1] Merikangas KR, He JP, Burstein M, Swanson SA, Avenevoli S (2010). Lifetime prevalence of mental disorders in U.S. adolescents: results from the National Comorbidity Survey Replication--Adolescent Supplement (NCS-A). J Am Acad Child Adolesc Psychiatry.

[2] van Veen JF, van Vliet IM, Derijk RH, van Pelt J, Mertens B (2008). Elevated alpha-amylase but not cortisol in generalized social anxiety disorder. Psychoneuroendocrinology.

[3] Granger DA, Weisz JR, McCracken JT, Ikeda SC, Douglas P (2006). Reciprocal influences among adrenocortical activation, psychosocial processes, and the behavioral adjustment of clinic-referred children. Child Dev.

[4] Granger DA, Kivlighan KT, el-Sheikh M, Gordis EB, Stroud LR (2007). Salivary alpha-amylase in biobehavioral research: recent developments and applications. Ann N Y Acad Sci.

[5] Weiner H (1992). Perturbing the organism: The biology of stressful experience.

[6] Buchanan TW, Bibas D, Adolphs R (2010). Salivary α-amylase levels as a biomarker of experienced fear. Commun Integr Biol.

[7] Martel FL, Hayward C, Lyons DM, Sanborn K, Varady S (1999). Salivary cortisol levels in socially phobic adolescent girls. Depress Anxiety.

[8] Stroud LR, Foster E, Papandonatos GD, Handwerger K, Granger DA (2009). Stress response and the adolescent transition: performance versus peer rejection stressors. Dev Psychopathol.

[9] Allwood MA, Handwerger K, Kivlighan KT, Granger DA, Stroud LR (2011). Direct and moderating links of salivary alpha-amylase and cortisol stress-reactivity to youth behavioral and emotional adjustment. Biol Psychol.

[10] Out D, Granger DA, Sephton SE, Segerstrom SC (2013). Disentangling sources of individual differences in diurnal salivary alpha-amylase: Reliability, stability, and sensitivity to context. Psychoneuroendocrinology.

[11] Adam EK, Till Hoyt L, Granger DA (2011). Diurnal alpha amylase patterns in adolescents: associations with puberty and momentary mood states. Biol Psychol.

[12] Gordis EB, Granger DA, Susman EJ, Trickett PK (2008). Salivary alpha amylase-cortisol asymmetry in maltreated youth. Horm Behav.

[13] Susman EJ, Dockray S, Granger DA, Blades KT, Randazzo W (2010). Cortisol and alpha amylase reactivity and timing of puberty: vulnerabilities for antisocial behaviour in young adolescents. Psychoneuroendocrinology.

[14] Lu Q, Tsao JC, Myers CD, Kim SC, Zeltzer LK (2007). Coping predictors of children’s laboratory-induced pain tolerance, intensity, and unpleasantness. J Pain.

[15] Lu Q, Zeltzer LK, Tsao JC, Kim SC, Turk N (2005). Heart rate mediation of sex differences in pain tolerance in children. Pain.

[16] Tsao JC, Lu Q, Myers CD, Kim SC, Turk N (2006). Parent and child anxiety sensitivity: relationship to children’s experimental pain responsivity. J Pain.

[17] March JS, Parker JD, Sullivan K, Stallings P, Conners CK (1997). The Multidimensional Anxiety Scale for Children (MASC): factor structure, reliability, and validity. J Am Acad Child Adolesc Psychiatry.

[18] Anderson ER, Jordan JA, Smith AJ, Inderbitzen-Nolan HM (2009). An examination of the MASC Social Anxiety Scale in a non-referred sample of adolescents. J Anxiety Disord.

[19] Tanner JM (1962). Growth at adolescence: with a general consideration of the effects of hereditary and environmental factors upon growth and maturation from birth to maturity.

[20] Tanner JM, Gardner LJ (1975). Growth and endocrinology of the adolescent. Endocrine and diseases of childhood.

[21] Gragg RA, Rapoff MA, Danovsky MB, Lindsley CB, Varni JW (1996). Assessing chronic musculoskeletal pain associated with rheumatic disease: further validation of the pediatric pain questionnaire. J Pediatr Psychol.

[22] McGrath PA, Gillespie J, Turk DC, Melzack R (2001). Pain assessment in children and adolescents. Handbook of pain assessment.

[23] Miller A, Barr RG, Young SN (1994). The cold pressor test in children: methodological aspects and the analgesic effect of intraoral sucrose. Pain.

[24] Out D, Bakermans-Kranenburg MJ, Granger DA, Cobbaert CM, van Ijzendoorn MH (2011). State and trait variance in salivary α-amylase: a behavioral genetic study. Biol Psychol.

[25] Bosch JA, Veerman EC, de Geus EJ, Proctor GB (2011). α-Amylase as a reliable and convenient measure of sympathetic activity: don’t start salivating just yet!. Psychoneuroendocrinology.

[26] McLaughlin KA, Kubzansky LD, Dunn EC, Waldinger R, Vaillant G (2010). Childhood social environment, emotional reactivity to stress, and mood and anxiety disorders across the life course. Depress Anxiety.

